# “Living Well” in the Constitution of Bolivia and the American Declaration on the Rights of Indigenous Peoples: Reflections on Well-Being and the Right to Development

**DOI:** 10.3390/ijerph17082870

**Published:** 2020-04-21

**Authors:** Karen Giovanna Añaños Bedriñana, Bernardo Alfredo Hernández Umaña, José Antonio Rodríguez Martín

**Affiliations:** 1 Department of Constitutional Law and the Institute of Peace and Conflicts (IPAZ), University of Granada, 18001 Granada, Spain; karengananos@ugr.es; 2 Faculty of Sociology and the Institute of Peace and Development (IPAZDE), Santo Tomas University, Bogota 110231, Colombia; bernardo.hernandez@usantotomas.edu.co; 3 Department of Applied Economics, University of Granada, 18071 Granada, Spain

**Keywords:** American Declaration on the Rights of Indigenous Peoples, Bolivia, development, indigenous peoples, living well, mother earth, well-being

## Abstract

The article analyzes how approaches to “Living Well” as reflected in the Constitution of the State of Bolivia, the Law of the Rights of Mother Earth, and the American Declaration on the Rights of Indigenous Peoples of the Organization of American States (OAS) contribute to understanding the Andean cosmovision of indigenous peoples of the American continent. To do so, it first studied the most immediate precedents that led to incorporation of the notion of Living Well into Bolivian law. Second, it approached the right to development from the American Declaration on the Rights of Indigenous Peoples, which has as its source the United Nations Declaration of the Rights of Indigenous Peoples. The paper thus proposes reflections on the Bolivian State and the American Declaration that advance understanding of Living Well, a notion comparable in the West to the right to development (political, social, economic, environmental, and cultural) that enables the individual and collective realization of the individual. Fullness, understood in terms of well-being, is related to the protection of health and of the environment. Finally, the paper employs a qualitative methodology with a well-documented hermeneutic focus, as well as the tool of a semi-structured interview with a Bolivian scholar familiar on the topic.

## 1. Introduction

In 2017, the Economic Commission for Latin America and the Caribbean (ECLAC) counted 826 indigenous peoples, of which 200 live in voluntary isolation. In 2014, Bolivia had the largest proportion of indigenous persons in its total population of any country in the region, 62.2% or over 6 million people. Like Bolivia, other ECLAC countries have large numbers of indigenous inhabitants relative to their total population: Guatemala 41% or 5.9 million, and Peru 24% or 7 million [1]. As these territories are home to the largest indigenous populations on the American continent, Bolivia, like Ecuador, Brazil, Colombia, and Mexico, can contribute considerably to building intercultural dialogue motivated by a culture of coexistence.

By way of comparison, Bolivia’s National Statistics Institute (INE) cites the country’s population as 11.5 million [2]. According to Bolivia’s Plurinational Communication Agency (APC), 84% of the population speaks (Castilian) Spanish, 28% Quechua, 18%Aymara, and 1% Guaraní [3]. Along with Spanish, all languages of indigenous nations and peoples are recognized as official languages. This policy means that nearly 50% of Bolivia’s total inhabitants speak an indigenous language.

At the political–governmental level, the region has had only two presidents of indigenous origin, that of Benito Pablo Juárez García in Mexico and, more recently, Juan Evo Morales Ayma in Bolivia. At judicial level, Bolivia has one of the most advanced constitutions [4] internationally with recognition and legal protection of indigenous persons and their populations. Bolivia also has specific legislation that protects these populations, the land, territory, and natural resources, referred to collectively in everyday parlance as“Mother Earth.” Ecuador and its Constitution follow similar guidelines.

These issues make Bolivia an ideal country for this study compared to the other Andean countries in South America (e.g., Peru, Ecuador, Colombia, Venezuela, Argentina, and Chile), as Bolivia has made a significant contribution to understanding Living Well, or, in Ecuador’s terms, Good Living.

This study demonstrates the dynamics of complementarity between international law and Bolivian national law. Within this conceptual framework, the Results section pursues two main focuses. The first is a documentary, judicial, national, international, and legal study. Its purpose is, on the one hand, to understand the social context of indigenous persons and their peoples in Bolivia and the main reasons for their continual vindications due to lack of a sufficiently inclusive policy on the part of the State; and, on the other, to conceptualize Living Well from the Andean cosmovision. We then examine Bolivian law as proof that it is possible to protect these vulnerable populations legally by including their rights and freedoms, the rights connected to Living Well (right to one’s territory, to land, to water, to health, to natural resources, to care for biodiversity, to a clean and healthy environment, to peace, to harmonious social relations, to social justice, to climate justice, to dialogue of knowledge, to solidarity among human beings, to complementarity and equilibrium, etc.). This idea also leads to the inclusion of other inherent principles, such as “amaqhilla” (do not be lazy), “amallulla” (do not be a liar), and “amasuwa” (do not be a thief), in the legal instrument par excellence of the entire democratic State, the Constitution. 

The second focus of this study is the “right to development” of the American Declaration on the Rights of Indigenous Peoples [5], which is a constituent element of Andean Living Well. This right is, however, compatible with and complementary to other rights (to identity and integrity, spirituality, health, protection of the healthy environment, peace, security, protection, etc.), in the American Declaration. These rights had previously been included in the United Nations Declaration of the Rights of Indigenous Peoples [6], which encompasses the whole of Andean Living Well; that is, the concept of Living Well, understood in the West in terms of Development and Well-Being. 

All of this theoretical framework of results enables us in the Discussion section to analyze the points of agreement and disagreement—first within the understanding of Living Well from a Bolivian perspective, and, second, from an international perspective—in order to establish the contributions of Bolivian national and American international law. Finally, we stress the importance of indigenous people and care for Mother Earth. This importance is closely tied to the environment as it relates to Andean Living Well, or knowing how to live, which is synonymous with health and well-being.

In this sense, the study concludes that there are points of agreement between the rights included in the Bolivian Constitution and the American Declaration concerning the term “Living Well.” Although each document approaches the issue from different points of view, the ultimate purpose of both legal texts is to ensure the recognition, promotion, protection, and guarantee of the rights and freedoms of indigenous peoples. Along these lines, Article 2 of the Universal Declaration of Human Rights states that:

*“everyone is entitled to all the rights and freedoms set forth in this Declaration, without distinction of any kind, such as race, color, sex, language, religion, political or other opinion, national or social origin, property, birth or other status.”*

Finally, we note that the scenario is not encouraging for indigenous peoples on the American continent. According to the ECLAC Report, Social Panorama of Latin American in 2018, “Structural gaps in inclusion operate to the detriment of the rural population, women, young people, and indigenous and Afro-descendent persons.” Furthermore, ongoing problems such as “persistent inequality in access to well-being and in the exercise of political, economic, social and cultural rights” threaten not only the economy but also the most vulnerable groups, such as the indigenous community [7]. In this context, predictions are that it will be difficult for the region to make global progress on the Agenda 2030 for Sustainable Development [8]. Bidding for equality of rights, opportunities, capabilities, and recognition is thus a tremendous challenge (as well as an obligation) in the medium and long term in Latin America. 

## 2. Materials and Methods

### 2.1. Case Study

We chose to study Bolivia and its domestic law due to their distinctive political, social, and cultural characteristics, but especially for their remarkable advance in protection and guarantees of the rights of indigenous peoples, Mother Earth, and Living Well, relative to other countries in this South American region. These countries are considered Andean primarily because their geographies share the Andes, the mountain range that runs along the western side of the American continent. Since the Andes extend from Venezuela to Argentina, we can speak of Andean or indigenous culture as a mix of the Hispanic from Spanish colonization and the indigenous shaped particularly by the Inca Empire.

### 2.2. Methods

For the development of the research, different methodological techniques have been used in each of the phases in which the article is structured, but it should be noted that the holistic and hermeneutic method has been followed fundamentally.

To achieve our goals, we grounded the study in a qualitative methodology [9,10,11,12,13,14,15]. Therefore, we work from “description and explanation of social phenomena and situations(…) to in-depth analysis and subjective interpretation of the phenomena and situations based on the discourse about them. This means examining not the externalities of human behavior but rather its logic of interiority” [16]. 

In particular, the study used data collected from descriptive sources and many observations that allowed us to analyze and identify key aspects of the concepts of Living Well in the Constitution of Bolivia, in relation to the right to development of the American Declaration of Indigenous Peoples. As stated above, this approach enables us to analyze the social, legal, and cultural context in order to explain why today, in the 21^st^ century, we must still fight for the protection of indigenous peoples and safeguard their cosmovision, that is, fight for Andean Living Well.

### 2.3. Documentary Analysis and Interview

Different qualitative techniques applied at different stages of the work were used throughout the research. Initially, data collection and documentary analysis were carried out largely during a research stay, in 2018, in La Paz, Bolivia. The main bibliographical sources, in relation to the idea of Living Well, were consulted at the University Mayor de San Andrés, in La Paz, and at the Autonomous University Gabriel René Moreno, in Santa Cruz de la Sierra.

Within this scenario, we performed documentary and bibliographic analysis of various primary, judicial, legal, constitutional, and international sources, as well as legal doctrine, concerning decisions of the Inter-American Court of Human Rights (hereafter, IHR Court). We also used material from scholarly articles and journals in the field, and from digital media (e.g., searching the Internet for electronic material and accessing a variety of current databases).

In a second phase, different qualitative techniques such as ethnography were used in the elaboration of our study, which were based on the observation resulting from the interpretation, and in a complementary way, a semi-structured interview (SI) was conducted [17,18,19]; this was carried out in the field work in La Paz, Bolivia, in 2018. For this, a prestigious professor was chosen, a Bolivian sociologist.

The professor was a member of the Diversity Network, the Latin American Network of Living Community Culture, and the Permanent Work Group on Alternatives to Development, in Alto de la Paz, Bolivia. The choice of the professor, a recognized specialist, was justified by his extensive professional experience with more than twenty years of work in the scientific and social dissemination around the cultural studies and the Living Well of indigenous peoples, based on social movements.

The questions of the semi-structured interview focused on the experiences of the participant to determine his perception of the origins of the discussion of the use and concept of Living Well, understood from the indigenous worldview in relation to the development model and practices of the West in terms of modernity.

Mainly, the professor highlighted the great importance of considering the Living Well of other forms of life that coexist in the world, such as the indigenous peoples of Bolivia, which invites the pursuit of a social, environmental, and spiritual internal balance and harmony, that will have an impact on our well-being. Therefore, this area of research is of high interest in regard to human rights and the rights of nature. It also highlights the relevance of the national and international normative development that has taken place in recent years, which addresses the protection and right to the development of indigenous peoples and the legal documents that have preceded it. Finally, the authors declare that there is no conflict of interest between researchers and other parties or financial support, nor has an ethical request been necessary, according to the methodology developed in the work.

## 3. Results

### 3.1. Bolivia and the Rights of Indigenous Peoples

After Bolivian independence (1825), vindication of the rights of native indigenous peoples in the history of Bolivia became a process limited to indigenous persons themselves and their excluded peoples. Yet their constant struggle helped them to achieve their goals gradually over time. Along this path, we stress the entry into force first of Convention 107 and then of Convention 169 of the International Labor Organization (ILO) [20]. For today’s Plurinational State of Bolivia, this international instrument has constituted a primarily legal and political tool to pressure governments (which were far from recognizing the rights of the indigenous peoples) and demand that the States fulfill their duty to guarantee these rights. 

From 1990 to the present, we count ten marches for vindication [21]. These protests made visible both the social discontent of the people and the successive political administrations’ unfulfilled promises. Led by the indigenous people of the lowlands and bringing mobilization throughout the national territory, these protests achieved changes that would not have been possible working in isolation. Indigenous peoples’ vindication of their territories, resources, and dignity was not easy and included episodes of violence. However, the State of Bolivia heard their outcry and responded in cases such as the Cochabamba Water War of February2000, the “Bolivian Gas War” of 2003, and the challenges to democratic procedures perpetrated by then-president Gonzalo Sánchez de Lozada (who served two terms, 6/08/1993 to 6/08/1997 and 6/08/2002 to 17/10/2003, after renouncing the presidency). The government also failed, however, to fulfil many of the promises agreed upon in the three immediately preceding marches in 1990, 1996, and 2000, respectively. The political goal of the fourth march in 2002 was thus to force the establishment of a Constituent National Assembly (inaugurated officially in August of 2007).

After a profound political crisis, as Marco Baldivieso writes, “Bolivia chose to transform the State and endow it with a new constitution. The ever conflict-ridden process of democratic transition—without physical violence—gave birth to the Plurinational Constitution, which synthesizes the emergence of new hegemonies and signified tremendous departures from traditional paradigms.” As a result, “great changes occurred in all areas” [22]. For example, in the 2005 elections, Juan Evo Morales Ayma, an indigenous rural leader, obtained 54% of the vote, taking office as the 60^th^ president of the Plurinational State of Bolivia and first president of indigenous origin, serving from 22 January 2006 to 10 November 2019.

### 3.2. Approaches to Andean Living Well 

Ecuador uses the term “Good Living” [23,24], known in Kichwa (Quechua) as Sumak Kawsay; Bolivia uses the term “Living Well,” whose official translation from the Aymara is Suma Qamaña, and other understandings appear in the Bolivian Constitution. In these two Andean countries, “a political and institutional development of these notions took place due to their inclusion in the countries’ respective political constitutions and has become a priority, not only on their agendas (since 2008 and 2009, respectively) but also among indigenous and non-indigenous scholars” [25].

Javier Medina authored one the most significant studies of Suma Qamaña [26]. For Medina, the “Amerindian” Andean cosmovision transcends the Western Newtonian, Cartesian, and reductionist-deterministic view. The Western cosmovision is based on the principle of non-contradiction, which comes from the principles of identity (A is A) and the excluded middle (a proposition is either true or false; there is no third possibility). These principles lead to the understanding that there is a single absolute truth, which translates into a rationality of exclusion in the social and political realm and tends toward developmentalism. 

In contrast, the “Amerindian” outlook can be understood from an inclusive perspective. When faced with duality, it sees instead a dual unity that holds multiple possibilities, as does Living Well. This view is based on two principles: the principle of complementarity of opposites (A and B are opposed but complement each other in a contradictory relation that completes them); and the principle of the included middle, which proposes a third possibility beyond the relation of contradiction, thereby enabling this third possibility to appear. In addition to these two principles, Medina includes the principle of reciprocity. Reciprocity involves establishing relationships as twofold in the order of the sacred, with both the cosmos and human beings, based on the understanding that life grows and knowing is loving. Reciprocity thus involves understanding reality, emphasizing but not stopping to explain that this is the epistemological location of Suma Qamaña [26].

Similarly, in discussing the diverse perspectives of Living Well/Suma Qamaña in his interview, Professor Mario Rodríguez links the notion to the discussion of native indigenous peoples’ ways of life, which must be expressed and made visible. These ways of life are not inherently the same as those in the West (developmentalist model), but this does not mean we must return to the “age of cavemen.” On the contrary, these new perspectives have elements in common with bids for alternatives to developmentalism and are called Living Well because they recognize and rest on the pillars of coexistence involved in Living Well Together. 

Rodríguez also calls us to recognize that the idea of Living Well is not only a question for indigenous peoples. Rather, it seeks to reestablish relationships among human beings, between human beings and nature, and between the ancestral and the sacred. Practicing and experiencing it gives rise to equilibrium in relationships of reciprocity and horizontality. More specifically, Rodríguez tells us that Living Well is explained by analogy to Illa (that is, that which already is that which will be, without yet being what it already is), understood as what grows from the first plans of the chacra (a small plot of land or rural farm that includes a dwelling and land to raise plants and domestic animals). It is a horizon of meaning, not of arrival: we do not know how it ends because everything depends on how life grows.

Mario Torrez has discussed Living Well as part of a complementary duality based on the foundation of well-being. One must achieve equilibrium between the existential and the experiential, within and without, in home and community, and this is precisely what makes Living Well possible [27]. Simón Yampara holds that Living Well is a matter of integral harmony between living and living together. It also has to do not only with life, but also with death [28]. As David Choquehuanca indicates, Living Well is the harmony between the material and the spiritual [29]. It therefore involves living a full life, a notion to which Fernando Huanacuni refers to when arguing that knowing how to live is living in harmony and equilibrium with the cycles of Mother Earth, the cosmos, and all forms of life and existence. It follows that whoever knows how to live, knows how to live in a community, that is, to live together [30].

Huanacuni differentiates between Living Well and Living Better. Living Well is associated with living in community, that is, in complementarity, reciprocity, harmony, and solidarity. Living Better is connected to the current model of development, determined by the capitalist economic system, which encourages individualism, disequilibrium, consumerism, and competition, a relational asymmetry, and to excessive anthropocentrism. 

This approach invites us to rise to a level that enables reunion among human beings and non-human beings. According to Xavier Albó, Living Well also involves native indigenous peoples affirming their existence in promoting the culture of life, understood not only from the human but also with the natural. Ultimately, they stress the importance of caring for and nurturing our existence, which Albó understands as Suma Qamaña Living (Together) Well, because it involves the ethical and the spiritual and valuing and appreciating the other, which is different [31]. In essence, Living Well is living in peace and living at ease. It means the good life, and it is nurturing life with affection [32]. 

In this context, we must introduce another expression from the current Bolivian Constitution, Teko Kavi, which the Guaraní people understand as Living Well. Professor Víctor Villavicencio translates Teko Kavias “the true way of being, which, rather than being an ideal of life is a dynamic state of continuous transformation and approach to community with our Father Nanderuvusu,” in Guaraní synonymous with God [33]. 

Along these lines, Professor Fernando Heredia holds that “Teko Kavi is a paradigm of life for the Guaraní people of Bolivia, a social construct that explains the distinctive way of understanding human and natural life in its various dimensions.” By extension, Teko Kavi is formed of three space-times. The first is the act of creation, which grounds the divine condition of humans and establishes the good way for human beings to be and relate to each other and to nature. The second shapes the relationship between the land and territory, seen not as Mother Earth but as sister and brother. This notion has to do with the place in which one is and gives rise to Teko Kavi, and thus to the complementarity of opposites, forging a dialectic that balances different forces. The third is the locus of what is simply called earth, as the place of plenitude and perfection of the human [34].

Despite the different interpretations of Suma Qamaña and Teko Kavi, we see their consistent connection to the human and non-human, the sacred and the natural. They go beyond rationality and Newtonian determinism, promoting exploration of new paths that feed discussions about other ways of living and alternatives to developing mentalist discourses. They do so despite criticisms of this way of living and living together as utopian and unrealizable for such a complex humanity, and despite attribution of the authorship of Living Well to native indigenous peoples [34].

In the words of Alison Spedding, this does not mean that this notion does not exist or that it cannot be carried out, much less that it is invalidated as an irrational position. For if one thing has been proven, it is that processes of change takes time and only develops with the participation of those that have historically been excluded, or who have decided to leave their comfort zone. The latter is the case of Bolivia’s native indigenous peoples, whose marches and vindication of their rights and human dignity have brought about a change in their Constitution, a change that brings greater protection of the rights and freedoms of the most vulnerable populations [35].

### 3.3. Living Well in Bolivian Law 

#### 3.3.1. Living Well in the Constitution of Bolivia

Living Well figures prominently in the new 2009 Constitution of Bolivia [4]. Of its 411 articles, over 30 relate directly to rural native indigenous populations and correspond to these populations’ civil, economic, social, political, cultural, environmental, and territorial rights, as well as to their autonomy, indigenous jurisdiction, etc. 

One of the characteristics of the Constitution “is the explicit addition of axiological content from indigenous cultures, such as values and principles.” These values and principles are included in the Preamble, among them, “sovereignty, dignity, complementarity, solidarity, harmony, and equity in the distribution and redistribution of the social wealth, where the search for the good life of Living Well predominates, with respect for plurality” [22].

The influence of the Andean vision and the experience of the people of the lowlands (the Amazon basin, which covers approximately two thirds of Bolivia) are present in the Preamble and throughout the text of the Constitution. Living Well thus forms one of the prioritized “axes” of the new State of Bolivia. Article 1 of the Constitution establishes its foundation as plurality and pluralism. Article 2 states that:

*“Given the pre-colonial existence of nations and rural native indigenous peoples and their ancestral control of their territories, their free determination, consisting of the right to autonomy, self-government, their culture, recognition of their institutions, and the consolidation of their territorial entities, is guaranteed within the framework of the unity of the State, in accordance with this Constitution and the law.”*

According to the Constitution, the nation of Bolivia includes “all Bolivians, the native indigenous nations and peoples, and the inter-cultural and Afro-Bolivian communities” (Article 3). Article 3 is inclusive of cultural diversity and gives clear recognition of disadvantaged populations. It also recognizes as official languages (in addition to Spanish), “Aymara, Araona, Baure, Bésiro, Canichana, Cavineño, Cayubaba, Chácobo, Chimán, EseEjja, Guaraní, Guarasu’we, Guarayu, Itonama, Leco, Machajuyai-kallawaya, Machineri, Maropa, Mojeño-trinitario, Mojeño-ignaciano, Moré, Mosetén, Movima, Pacawara, Puquina, Quechua, Sirionó, Tacana, Tapiete, Toromona, Uru-chipaya, Weenhayek, Yaminawa, Yuki, Yuracaré, and Zamuco” (Article 5.I).

Having presented the Bolivian model of the State, we will now focus on how the notion of Living Well is incorporated into Article 8 of the Constitution of Bolivia. First, Living Well is institutionalized (Article 8.I) such that the State assumes and promotes ethical–moral principles of ancestral origin, known in their original languagesas “suma qamaña” (live well), “ñandereko” (live harmoniously), “teko kavi” (good life), “ivi maraei” (land without evil) and “qhapaj ñan” (noble path or life). The Article also includes well-known precepts adopted by Quechua-/Kichwa-speaking peoples: do not be lazy, do not be a liar, and do not be a thief. 

Second, the State draws support from the values of “unity, equality, inclusion, dignity, liberty, solidarity, reciprocity, respect, interdependence, harmony, transparency, equilibrium, equality of opportunity, social and gender equality in participation, common welfare, responsibility, social justice, distribution and redistribution of the social wealth and assets for wellbeing (Living Well).” These are the values and principles that make up Living Well (Article 8.II).

The goal or basic functions of the State are (among others): “To guarantee the welfare, development, security and protection, and equal dignity of individuals, nations, peoples, and communities” (Article 9.2); to guarantee access to education, health, and work (Article 9.5); and “To promote and guarantee the responsible and planned use of natural resources, … as well as to preserve the environment for the welfare of present and future generations” (Article 9.6).

Bolivian legislators institute protection for rural native indigenous peoples, strengthened, in the words of Baldivieso, as “positive or affirmative action.” [22] They grant them “free determination, their territory, their institutions, their jurisdiction, their collective rights, communal property, indigenous regional governments, and special subdivisions of political representation,” as a form of reparations for all the centuries of marginalization, exclusion, and discrimination [36].

The Constitution reinforces this protection, as seen in native indigenous jurisdiction, which is granted constitutional scope and equal status with ordinary jurisdiction (Article 179.II). The legislators recognize the rural native indigenous population as “nation,” understood as “every human collective that shares a cultural identity, language, historic tradition, institutions, territory and world view, whose existence predates the Spanish colonial invasion” (Article 30.I). The second section of Article 30. IIlists 18 specific rights exclusive to these peoples, such as the right:

*“(…) 2. To their cultural identity, religious belief, spiritualties, practices and customs, and their own world view. (…)*

*9. That their traditional teachings and knowledge, their traditional medicine, languages, rituals, symbols and dress be valued, respected, and promoted.*

*10. To live in a healthy environment, with appropriate management and exploitation of the ecosystems. (…)”*

The10^th^ right to the environment has additional constitutional clauses to ensure its exercise (Articles 33 and 34). These clauses are connected to Title II, on Environment, Natural Resources, Land, and Territory, in which the State and the Bolivian population are made responsible to uphold their duty “to conserve, protect and use natural resources and biodiversity” (Articles 342 to 347). Environmental management policies are established to achieve this goal, since this common wealth and heritage are of public interest. The fundamental human right to water receives similar treatment (Articles 373 to 377). Ultimately, the State of Bolivia is directly responsible for guaranteeing, protecting, and respecting the rights of indigenous nations and peoples (Article 30.III). These rural native indigenous nations and populations can thus exercise “their jurisdictional functions and competency through their authorities, and shall apply their own principles, cultural values, norms and procedures” (Article 190).

#### 3.3.2. Living Well in Legislation

In legislation on the rights of “Mother Earth,” we recall that the World People’s Conference on Climate Change and the Rights of Mother Earth approved the Universal Declaration of the Rights of Mother Earth [37], which established April 22 as Earth Day. The Declaration recognizes that “Mother Earth is the source of life, nourishment and learning and provides everything we need to live well.”

Bolivia ratified the Declaration on October 12, 2012, and ex-president Evo Morales’ government promoted it through promulgation of a series of laws to implement application of the Declaration of the Rights of Mother Earth and enable the process of change toward Living Well and respect for Mother Earth. These include the following laws:

1. Law 071 of 21 December 2010 [38]. One of the principles of the “Law of the Rights of Mother Earth” is respect for and defense of the rights of Mother Earth. This principle states that “The State and any individual or collective person must respect, protect, and guarantee the rights of Mother Earth for the well-being of current and future generations” (Article 2). Mother Earth is thus “considered sacred, from the worldviews of nations and rural indigenous peoples” (Article 3). Finally, this law establishes the responsibility and duty of the State and society to guarantee the right to life, diversity of life, water, clean air, equilibrium, and restoration and pollution-free living (Article 7).
2. Law 300 of 15 October 2012. “Framework Law of Mother Earth and Integral Development for Living Well” [39]. Determines the legal framework and foundations for integral development in harmony and equilibrium with Mother Earth for Living Well (Article 1). This law is the main legislation regulating Living Well, with a total of 58 articles. The principles that govern it are: compatibility and complementarity of rights, responsibilities, and duties; non-commercialization of the environmental functions of Mother Earth; integrality; precautionary action; guarantee to restore Mother Earth; guarantee to regenerate Mother Earth; historical responsibility; priority of prevention; plural participation; water for life; solidarity among human beings; harmonious relation; social justice; climate justice; plural economy; complementarity and equilibrium; and dialogue of traditional know ledges and science (Article 4).
3. Values for Living Well interrelated with knowledge are established for the construction of a just, equitable, and solidary society. Living Well is knowing how to grow (in the framework of respect for freedom of religion and spiritual beliefs, according to the indigenous cosmovision); knowing how to feed oneself (with quality and natural produce, respecting the seasons of the year and foods); knowing how to dance (dancing in gratitude to Mother Earth to express spirit and energy); knowing how to work (considering work as celebration and happiness expressed with love and passion); knowing how to communicate (feeling and thinking to speak well and contribute); knowing how to dream (of a good future, projecting life); knowing how to listen (to know ourselves, recognize ourselves, respect ourselves, and help ourselves; listening to elders to revalue ancestral knowledges); and knowing how to think (not only rationally but also from feeling). These values are included in Article 6.
4. Supreme Decree 1696 of 14 August 2013. “Regulation of the Framework Law of Mother Earth and Integral Development for Living Well,” whose goal is “to regulate the functioning of the High Plurinational Authority of Mother Earth.”

### 3.4. American Declaration on the Rights Related to Living Well

On 15 June 2016, after 27 years of joint work by the Organization of American States (OAS), non-governmental organizations (NGOs), civil society, and representatives of indigenous populations, the General Assembly adopted the American Declaration on the Rights of Indigenous Peoples (hereafter, OAS Declaration) [25,40,41,42,43].This declaration draws especially on the 2007 United Nations Declaration of the Rights of Indigenous Peoples (hereafter, UN Declaration). 

The UN Declaration is one of the broad international instruments compared to other instruments concerning minorities (currently, 148 countries support the UN Declaration). Its value lies in its defense and protection of indigenous peoples as permanent societies, and its respect for ethnic and cultural diversity. The UN Declaration also ratifies the rights to identity, culture, language, systems of writing and literature, oral traditions, health, work, education, and other rights in the ILO Convention. Most significantly, it marks a watershed in that it provides a shared framework for improving temporary situations where two opposed interests collide, that is, the rights of indigenous peoples and State politics. 

The UN Declaration thus constitutes a precedent of international law in its recognition, protection, and promotion of the rights of indigenous peoples that provides measures for State action when problems of indigenous populations arise [44]. In this context (in addition to collective rights, which are indispensable to the existence and well-being of these populations), it stresses free determination of peoples, the right to land, territory, and resources, economic, social, and cultural rights, and equality and nondiscrimination.

Although negotiations to adopt the OAS Declaration by consensus without reservations or observations continued until the last moment, such adoption did not occur. Countries such as the United States of North American, Canada, Colombia, and Brazil imposed their own observations, linked to topics such as free determination, consultation, and consent [45,46,47,48], collective rights, and natural resources, land, and territories [49,50]. The OAS Declaration also recognizes the importance of the existence of indigenous peoples and their tremendous contribution to development, plurality, and cultural diversity in the societies of the American continent, despite the historical discrimination and marginalization they have suffered and continue to suffer from the colonial era to our time (Preamble of the OAS Declaration).

The OAS Declaration is an international instrument that strengthens the relationship between States and indigenous peoples of the American continent, where the main criterion is collective and individual self-identification. The OAS Declaration has 41 articles on rights, the participation of indigenous peoples, and contributions of indigenous legal and organizational systems. Finally, Article 41 of the OAS Declaration states that “The rights recognized in this Declaration and the United Nations Declaration on the Rights of Indigenous Peoples constitute the minimum standards for the survival, dignity, and well-being of the indigenous peoples of the Americas.”

#### 3.4.1. Right to Development

Living Well is comparable to the right to development in the OAS Declaration, which finds its precedent in the UN Declaration on the Rights of Indigenous Peoples [6] and the UN Declaration on the Right to Development [51]. The latter defines:

“the right to development [as] an inalienable human right by virtue of which every human person and all peoples are entitled to participate in, contribute to, and enjoy economic, social, cultural and political development, in which all human rights and fundamental freedoms can be fully realized.”

These are the claims for the individual. In parallel, from the collective perspective, development implies “the right of peoples to self-determination which includes, subject to the relevant provisions of both International Covenants on Human Rights, the exercise of their inalienable right to full sovereignty over all their natural wealth and resources” (Article 1). “The human person is the central subject of development and should be the active participant and beneficiary of the right to development” (Article 2). 

Various legal instruments on indigenous issues have considered the right to development in their different articles. First, Convention 169 (Articles 7 and 23) of the ILO includes the right of indigenous peoples to take decisions about priorities concerning the process of development and the duty of States to lend their support (Article 7). The Convention also stresses the importance of the traditional activities of indigenous peoples to their development (Article 23) [20].

Second, through the principle of free determination, the UN Declaration on the Rights of Indigenous Peoples recognizes “political, economic, social, and cultural development. And to end all forms of discrimination and oppression, wherever they occur.”These peoples should control the events that affect them and their lands, territories, and resources according to their needs and aspirations (Preamble). States must therefore adopt effective measures such as free, prior, and informed consent concerning these lands and territories (Article 29) [6].

The OAS Declaration also materializes the right to development in Article 29. The content of this article is very similar that established by the UN Declaration mentioned above. More specifically, this article includes five situations:

1. Indigenous peoples have the right to maintain and determine their own priorities with respect to their political, economic, social, and cultural development in conformity with their own cosmovision. They also have the right to be guaranteed the enjoyment of their own means of subsistence and development, and to engage freely in all their economic activities [4,6].
2. This right includes the “development of policies, plans, programs, and strategies” for the exercise of their right to development and to implement them in accordance with their legal, political, social, institutional structure and their cosmovisions.
3. Indigenous peoples have the right to be actively involved in developing and determining development programs that affect them and, to the extent possible, to administer such programs through their own institutions and their cosmovision.
4. States shall consult and cooperate in good faith with the indigenous peoples concerned through their own representative institutions in order to obtain their free and informed consent [52,53] prior to the approval of any project affecting their lands or territories and other resources, particularly in connection with the development, utilization or exploitation of mineral, hydric [“water”], or other resources) [54]. The State shall thus hold “consultations and cooperate in good faith with indigenous peoples.”
5. Indigenous peoples have the right to effective measures to mitigate adverse “ecological, economic, social, cultural, or spiritual impacts of the implementation of development projects” that affect their rights. Indigenous peoples who have been deprived of their means of subsistence and development have the right to restitution and, where this is not possible, to fair and equitable compensation. This includes the right to compensation for any harm caused to them by the implementation of plans, programs, or projects of the State, international financial institutions, or private business.

It is also important to mention briefly the legal development of the IHR Court on this issue. The IHR Court was a pioneer in expounding the close relationship of indigenous and tribal peoples to the land. Specifically, the IHR Court recognized the right of indigenous and tribal peoples to enjoy the earth without need of a formal title to property. It has decreed the adoption of special measures to guarantee exercise of the right to protection of physical and cultural survival, as in Mayagna (Sumo) Awas Tingni Community v. Nicaragua [55]. Similarly, in the case of the People of Sarayaku. Ecuador [56], the Court recognized “the deep cultural, non-pecuniary and spiritual tie that the community has with its territory.”

Further, the IHR Court stipulates indigenous and tribal peoples’ right to property in the case of natural resources discovered in their territories. Without resources, the economic, social, and cultural survival of the peoples is at risk, as demonstrated in the case of Yakye Axa Indigenous Community v. Paraguay [57]. Similarly, lack of access to and enjoyment of natural resources condemned the Xakmók Kásek people [58] to live in conditions of poverty and marginalization. According to Jorge Calderón, indigenous peoples and natural resources “constitute more than a dialectical relationship. It is impossible to conceive of the existence of one without the other” [59]. The following cases exemplify these issues: Awas Tingni Community v. Nicaragua [55]; Yakye Axa Indigenous Community v. Paraguay [57]; Saramaka People v. Suriname [60]; Xákmok Kasek Indigenous Community v. Paraguay [58]; People of Sarayaku v. Ecuador [56], and Kaliña and Lokono Peoples v. Suriname [61].

On this order of ideas, the IHR Court also identifies the tremendous importance of the right to consultation and prior, free, and informed consent. The right of indigenous peoples to participate and to have their institutions and cosmovision respected includes the definition, demarcation, and allocation of a title to an indigenous or tribal territory, taking into account “traditional law, values, uses, and customs.” This assumption is reflected in Awas Tingniv. Nicaragua [55]. In Saramaka People v. Suriname, the Court recognized that “the members of indigenous and tribal peoples have the right to own natural resources that they have used within their traditionally owned territory.” The Court admitted the right to protect land and the resources habitually used by indigenous populations to avoid their extinction. Thus, “it is very likely that extraction of a natural resource will affect the use and enjoyment of other natural resources necessary for survival” [60].

On the right to consultation, the Court determined in the case People of Sarayaku v. Ecuador that States have the obligation to consult with indigenous peoples. It based its decision not only on support for this notion in conventional law, but on also on the fact that the law of consultation constituted a general principle of international law that has been incorporated into domestic statutes and the jurisprudence of the great majority of the OAS member States [56].

Finally, it is worth noting that, following the recent promulgation of the OAS Declaration for the International Day of the World’s Indigenous Peoples (August 9), the Court Reporters of the UN and the Inter-American Commission of Human Rights (ICHR) published a joint press release reaffirming indigenous peoples’ right to development, adding that a countries’ development “has been and continues to be driven at the expense of indigenous peoples of the continent.” Ultimately, the right to development, on the one hand, implies the right of all indigenous peoples to define their own development priorities, whether political, economic, social, or culture, according to their cosmovision; on the other, it challenges the States to guarantee that indigenous peoples enjoy their own means of subsistence and development and dedicate themselves freely to their economic activities [62].

#### 3.4.2. Other Connected Rights

The right to development cannot be interpreted in isolation from other rights that complement it and facilitate understanding it as a whole if we seek to equate the right to development to Andean Living Well, which goes beyond the satisfaction of needs for comfort or consumption in monetary terms. Two sets of rights follow. The first set, the rights established in the third Section on Cultural Identity, includes: the right to cultural identity and integrity (Article 13); the right to preserve, use, develop, revitalize, and transmit to future generations their own systems of knowledge, language, and communication (Article 14); the right to education, particularly of indigenous children, at all levels and in all forms, without discrimination (Article 15); the right freely to exercise their own spirituality and indigenous beliefs(Article 16); the right to preserve, maintain, and promote their own family systems (Article 17); collective and individual rights to the enjoyment of the highest attainable standard of physical, mental, and spiritual health;(Article 18); and the right to protection of a healthy environment (Article 19).

The second set encompasses the rights in the Fifth Section on Social, Economic, and Property Rights, including: the right to maintain and strengthen their distinctive spiritual, cultural, and material relationship with their lands, territories, and resources(Article 25); the right to remain in voluntary isolation or initial contact, and to live freely and in accordance with their cultures (Article 26); labor rights, such that States shall take all specific measures necessary to prevent, punish, and remedy any discrimination (Article 27); the right to protection of their tangible and intangible cultural heritage and intellectual property, including its collective nature, transmitted over millennia from generation to generation (Article 28); and the right to peace, security, and protection(Article 30).

We will now focus on the right to peace, which is linked most directly to the right to development. Previously, the UN Declaration on Development [51] had asserted that “international peace and security are essential elements for the realization of the right to development.” In this context, “resources released through disarmament measures should be devoted to the economic and social development and well-being of all peoples and, in particular, those of the developing countries.”

The UN Declaration of the Rights of Indigenous Peoples understands the right to peace as the suppression of military activities in lands or territories of indigenous peoples, unless there is a public reason to justify intervention. In any case, the State must consult before using indigenous lands or territories (Article 30.) The OAS Declaration later views this matter similarly to that of its UN predecessor, but includes more extensive content. According to the Preamble of the OAS Declaration, only:

*“recognition of the rights of indigenous peoples in this Declaration will foster harmonious and cooperative relations among States and indigenous peoples, based on the principles of justice, democracy, respect for human rights, nondiscrimination, and good faith.”*

In their totality, these factors enable harmonious coexistence, that is, realization of the right to peace. The Declaration also includes this right explicitly in Article 30, under the title, “Right to peace, security, and protection” of indigenous peoples, in which indigenous peoples are subject to protection under law. This formulation differs from the UN Declaration on the Rights of Indigenous Peoples, which considers indigenous persons as the subject of protection.

Article 30 also recognizes the right to autonomy of indigenous institutions; the right to protection and security in situations of war (domestic or international), according to international humanitarian law; and the fourth Geneva Convention of 1949 on the protection due civil persons in times of war. In addition, Protocol II of 1977 on the protection of the victims of armed conflicts, which is not international in character, recognizes these rights. In the case of armed conflicts, States must take appropriate measures to protect the human rights, institutions, lands, territories, and resources of indigenous peoples and their communities.

Special emphasis is placed on protecting the most vulnerable in the group, such as indigenous children and women, prohibiting their recruitment in the armed forces, providing effective compensation and reparations for the harm or damage caused by an armed conflict, taking special and effective measures to avoid all forms of violence (especially sexual),simultaneously guaranteeing the right of access to effective justice, protection, and reparation of the damages caused to the victims, and protecting their right not to engage in military activities.

Within this scenario, the last report of the Inter-American Commission on the situation of indigenous peoples of the Amazon states that “Indigenous peoples have the right to be protected from forced displacement due to violence, as usually occurs in contexts of armed conflict.” By analogy, forced displacement directly attacks the existence of indigenous peoples due to their bond with their territory, “on which they depend for their physical and cultural survival.” The indigenous population displaced in urban environments is easy prey to poverty and discrimination, labor exploitation, sexual violence, human trafficking, and crime. For these reasons, States should adopt special protective measures, although such measures do not exclude taking measures to enable the return of indigenous people “to their traditional territories safely and with dignity” [63].

Finally, the right to international peace and security are fundamental elements for constructing the right to development. The right to peace and the right to development are closely related to Andean Living Well, which are related to the individual and collective harmony of indigenous persons and their peoples and, above all, to living together in and for peace.

## 4. Discussion

### 4.1. Contributions of National Bolivian Law and International American Law on the Rights of Indigenous Peoples to Living Well

As previously mentioned, Bolivia plays an important and critical role in contributing to issues concerning indigenous peoples, and this contribution spans the legal, political, economic, cultural, and environmental spheres. This section focuses on the legal sphere.

First, Bolivia has taken an important step in including in its Constitution [4] the prohibition and punishment of discrimination against indigenous persons and their populations (Article 14.II). As Bolivia is a country with a majority indigenous population, this is a crucial clause in the Constitution if advances are to continue in decreasing economic, social, and cultural gaps and fostering equality. This clause led to Law 045 of 8 October 2010, “Law against Racism and Any Form of Discrimination,” [64] which prohibits barring any Bolivian from establishments open to the public and requires visible posting of a sign that states, “All persons are equal before the law.”

This law originated to remedy the former prohibition of indigenous people and animals from access to public establishments. Specifically, the Framework Law of Mother Earth stipulates that Living Well means that indigenous persons live “in a just, equitable, and solidary society, without material, social, and spiritual poverty” (Article 4. Law 300).

Second, recognition of the following matters in the Constitution of Bolivia [4] provides some mechanisms to promote, protect, and guarantee the rights and freedoms of rural native indigenous populations:

1. A catalog of constitutional rights (Article 30.II).
2. The constitutional and hierarchical equivalence of ordinary and indigenous jurisdiction (Articles 190 to 192).
3. Recognition of rural native indigenous autonomy (Articles 289 to 296).

In fact, there must be compatibility and complementarity of “fundamental, civil, political, social, economic, and cultural rights to Living Well” (Article 4. Law 300). This idea includes individual and collective rights and freedoms linked to free determination of rural native indigenous peoples. But the concept is also associated with consultation and consent, natural resources, lands, and territory, which are often the subject of controversy between indigenous peoples and the State. According to Article 349 of the Constitution of Bolivia, natural resources are the property and direct and indivisible domain of the Bolivian people.

Third, on the right to development, Article 9 of the Constitution affirms that it is the goal and function of the State to guarantee the development, well-being, security, and equality of persons, nations, peoples, and communities. The State must therefore foster “mutual respect and intracultural, intercultural, and plurilingual dialogue.” Bolivia considers development to be integral, “in harmony and equilibrium with Mother Earth” (Article 1. Law 300), Integral Development for Living Well, understood as:

*“the continuous process of generating and implementing social, community, citizenly, and public management measures and actions for the creation, provision, and strengthening of conditions, capabilities, and material, social, and spiritual means within the framework of culturally sufficient and appropriate practices and actions”* (Article 5. Law 300).

Fourth, on the topic of Peace, the Constitution includes in Article 10 that Bolivia is “a pacifist State that promotes the culture of peace and the right to peace, as well as cooperation among the peoples of the region and the World…” (Article 10.I). Article 4 Section 12 of the Framework Law of Mother Earth speaks of the “harmonious, dynamic, adaptive, and balanced relationship” between the needs of Bolivia and Mother Earth. Peace is related to harmony, which means “Living Well amongst ourselves, Living Well with those around us, and Living Well with oneself.”

Finally, the influence of the Andean world view is present in the text of the Constitution. As Baldivieso writes: “Primary laws are enriched with rural native indigenous values and principles that generate fundamental and organic laws, but the law also keeps the higher goods inherent in Western culture; an interesting axiological symbiosis that gives rise to the development of Bolivian legal pluralism’’ [22].

Within this conceptual framework, we will now briefly tackle the contributions of the OAS Declaration. This is an international legal instrument, subsidiary to national law, and the State is the main organism responsible for and guarantor of protection of the rights of all of its citizens within its jurisdiction. As with the Constitution of Bolivia, one of the arguments for development of the OAS Declaration revolves around the vindication of indigenous and tribal populations, which were not historically included, but rather, were made victims century after century. The first checks were imposed on this situation at international level, with the OLI’s Convention 107 (ratified by Bolivia on January 12, 1965 and achieving automatic denunciation on December 10, 1992) and Convention 169(ratified by Bolivia, on December 11, 1991) [65]. Compliance with the Convention was compulsory. Subsequently, the UN Declaration and the OAS Declaration (cited above) were promulgated to ensure a minimum standard of protection of the individual and collective rights and freedoms of these fragile populations vis à vis the State.

The OAS Declaration recognizes that the vulnerability of indigenous and tribal populations led them to be dispossessed of their lands, territories, and resources, and that this has made it impossible for them to exercise their right to development, understood in terms of economic and social well-being. The Declaration recovers this respect for cultural identity and exercises the right to development according to one’s needs. Finally, it supports development as an essential element to achieve Living Well.

On the right to peace, the Declaration recognizes peace, along with security and protection, as a totality, in times of both peace and war or conflict (domestic or international) and draws on other international instruments and humanitarian international law. It seeks to protect its women, children, and adolescents, who are direct victims of conflicts (sexual trafficking or slavery). The Declaration also firmly rejects military activities in territories or lands of indigenous and tribal populations. The only exceptions are public interest and having reached an amicable agreement between the indigenous population and the State.

Definitely, the OAS Declaration attempts to equate indigenous peoples’ right to development and peace as integral elements of understanding Andean Good Living. Therefore, the right to development first recognizes the inseparable connection of indigenous peoples to the land, territory, and resources, which are conceived as a pillar of development. Second, the right to peace—defined as peace, security, and protection of indigenous peoples—incorporates essential elements for achieving the right to development. That is, both rights (peace and development) and other connected complementary rights (to their cultural identity and integrity; to their own spirituality and beliefs; to physical, mental, and spiritual health; to care of the environment; to strengthening their spiritual, cultural, and material relation to their land, territories, and resources; to remaining in isolation or initial contact, etc.) constitute the condition “sine qua non” to make it possible to speak in terms of equivalents to Andean Living Well. At the same time, these rights are mechanisms to guarantee the protection and defense of indigenous and tribal peoples against State decisions that may go against their economy, politics, culture, environment, or institutions.

Finally, the jurisprudence of the IHR Court plays a valuable role, first as the “cement” for establishing and consolidating the edifice of the rights of indigenous and tribal peoples, constructed primarily around the right to development and peace, both of which are intimately related to Andean Good Living. The Court also establishes that control and possession of land, territories, and natural resources are indispensable parameters for the survival and development of indigenous peoples. Further, it consolidates this inseparable association of indigenous populations and the Earth, their territories and natural resources (minerals, hydric (“water”) resources, energy, flora, fauna, etc.) as elements crucial to their economic, political, social, cultural, and institutional development from their Andean cosmovision. Finally, the Court seeks to construct the inseparable synergy between indigenous and tribal peoples and Mother Earth known as “Pacha Mama, Mucane, Tonanzín, Iwi, Nana Tlalli, Gaia” [57].

Appreciating and illustrating this close relationship that indigenous peoples have had historically with the environment and recognizing their rights are essential for the global implementation, monitoring, and fulfillment of the 17 Sustainable Development Goals (SDGs) in the Framework of the UN’s Agenda 2030 [8] along the lines of the UN Declaration on the Rights of Indigenous Peoples. The Agenda even makes specific reference to indigenous people small-scale agricultural production of indigenous farmers (Goal 2) and equal access to education for indigenous children (Goal 4). Finally, with the promise “that no one is left behind,” the SDGs acquire critical importance for the future and for respect of the rights of indigenous peoples.

### 4.2. Unfinished Business for theStates of the American ContinentConcerning the Care of the Environment

#### 4.2.1. The Inter-American Regional Agenda

At the Inter-American regional level, the Commission and Court for Inter-American Human Rights, as organisms for the protection of the Inter-American Human Rights System, have been working on indigenous issues and the environment. Since 2019, the IHR Court has handed down 24 sentences in favor of indigenous populations. Its legal work on protection of the environment remains, however, incomplete. The Inter-American Commission on Human Rights, in contrast, is already working on these issues and has produced three reports. These are the 2013 report on the situation of “Indigenous Peoples in Voluntary Isolation and Initial Contact in the Americas: Recommendation for the Full Respect of their Human Rights” [66]; the 2015 report on “Indigenous Peoples, Afro-Descendent Communities, and Natural Resources: Human Rights Protection in the Context of Extraction, Exploitation, and Development Activities” [67]; and the 2019 report on the “Situation of Human Rights of the Indigenous and Tribal Peoples of the Pan-Amazon Region” relating to environmental impact [63]. We discuss this last report briefly here.

We take the example of the Amazon Basin, part of which is located in Bolivia, with the rest distributed across eight other countries: Brazil, Colombia, Ecuador, Guyana, French Guiana, Peru, Suriname, and Venezuela (approximately 7 million km^2^). This area is considered the most biodiverse in the world, with tremendous cultural and biological wealth. It is also a “source of subsistence for the whole planet because it is a biome that operates as a global climatic stabilizer …and as a reserve of flora, fauna, and genetic diversity.” From the times of our ancestors to the present, this region has been inhabited and has served as the place where indigenous and tribal peoples develop their lives, knowledge, and traditional practices. It is home to around 350 peoples, including those in voluntary isolation and initial contact [63].

Although protection of the Amazon Basin—known as “the Pan-Amazon”—should be highest priority for the States’ regional and global agendas, it is at grave risk due to two environmental problems. The first is the desertification and deforestation of the Amazon rain forest; and the second is the loss of biodiversity and protected natural areas. Desertification can have grave environmental, social, political, and economic consequences. It directly influences the health of its inhabitants, not only of the Amazon region but of the world. Indigenous populations are the most affected, however, as they are forcibly displaced and lose their lands, which are dedicated to feeding them and to preserving their traditional medicine. Deforestation also risks causing the disappearance of peoples in voluntary isolation [63]. We reiterate that the principles of universality, human rights, equality, and sustainability of the environment are the highest priorities of indigenous peoples, who possess incalculable human capital in terms of traditional knowledge and the conservation of biodiversity.

#### 4.2.2. Bolivia and the Care for the Environment

The Bolivian Constitution’s Article 390.II states that the country’s Amazon territory includes the departments of Beni (provinces of Vaca Díez and Ballivián), Pando, La Paz (province of Iturrualde), Cochabamba and Santa Cruz [68], 724,000 km^2^ or 65.9% of Bolivia’s territory. According to the 2012 National Census of Population and Housing (CNPV) [69], there are 30 indigenous peoples in the Bolivian Amazon Basin. Currently, however, following the recent environmentally catastrophic fires in Brazil and affecting the countries of the Amazon Basin, this number has probably decreased. We still have no current definitive study of this serious deforestation.

Bolivia’s domestic law specifically includes the protection and preservation of the Amazon region at a Constitutional level (Articles 390, 391, 392). The State prioritizes the sustainable integral development of Bolivia for its “environmental sensitivity, existing biodiversity, hydric resources, and ecoregions.” The State also administrates this development, fostering the financing of tourism and ecotourism activities in coordination with the rural native indigenous peoples in the region. To do so, it facilitates dialogue through the creation of a special decentralized entity in the Amazon, fostering traditional extractive products. Finally, the State protects against cutting species such as the syringa and chestnut due to their “historic, cultural, and economic value,” declaring them symbols of the Bolivian Amazon. Violating this law leads to criminal punishment except in cases of public interest.

It is important to stress that Bolivia protects the environment as a right included in the Constitution (Article 30.II.10) and the laws favor Mother Earth and Living Well. Environmental law is protected by other constitutional clauses dedicated to its enforcement (Articles 33, 34). This right is also linked to the right to health, as the State further promotes “quality of life” free access of the Bolivian population to healthcare and protection of the traditional medicine of indigenous populations (Article 35).

These articles are also connected to Title II, on Environment, Natural Resources, Land, and Territory, which holds the State and the population responsible for the duty of preserving, protecting, and sustainably exploiting resources and biodiversity (Articles 342 to 347). To this end, environmental management policies are established, since this heritage is of public interest. There is also a special chapter on Hydric Resources. The right to water is recognized as “a most fundamental right for life” and health, through regulation of its use and integral management (fossil, glacial, medicinal, subterranean, and other waters; Articles 373 to 377).

Finally, we value very positively for a society like Bolivia its aspiration to collective and individual well-being of its citizens, reviewed in protection and guarantees of the right to the environment and its care. This strategy will be much more productive, however, if it not only includes national law but also makes the regional agenda of all States a priority, i.e., the agendas of both the American continent and the rest of the world. The first victims in this line of fire are always the most vulnerable, in this case, indigenous populations and those without contact or living in voluntary isolation, who live in harmony with nature and from whom we have much to learn. In fact, their participation and wisdom were fundamental elements in the processes that produced the UN’s Agenda 2030 for Sustainable Development [8].

### 4.3. Legal and Constitutional Conflicts over the Natural Resources of the Amazon and Indigenous Communities and their Traditions

#### 4.3.1. The Legislator Versus the Related Exploitation and Extraction Interests with the Natural Resources of the Amazon.

The Amazon has significantly affected the way of life, culture, and harmony with nature of the Amazonian indigenous peoples, who have been threatened since the twentieth century by increased extraction activities, reflected in increased logging, that accelerated deforestation that have given way to problems with other land uses. In recent times, there have been many changes, at the regulatory level, in State policies and practices aimed at increasing the extraction of natural resources and advancing large infrastructure projects, exerting pressure on indigenous ancestral territories [48,63].

In this context, there is a debate among States regarding the ratified international legal instruments of the United Nations and the Inter-American Organizations, emphasizing among other duties deriving from these instruments: (a) Design and effectively implementation of an adequate normative framework for the protection of human rights, which may be affected by extractive, exploitative and development activities; (b) the need for a legal framework that adequately addresses the operation of foreign companies within the jurisdiction of a State; and (c) the duty to prevent illegal activities and forms of violence against the population in areas affected by extractive activities, exploitation, or development [67].

Accordingly, the State must adopt the most appropriate legislation and not contradict these rights, for example, environmental protection standards are indispensable in domestic legislation, not to bring before the Court the State for violating the human rights of the affected populations, and for activities that affect the environment [67]. In this regard, the right to free, prior, and informed consultation and consent is a guarantee against infringement of this right.

The great contradiction between theory and practice makes it difficult for legislators to carry out their work and makes legal implementation difficult, in relation to the exploitation and extraction of the natural resources of the Amazon region, with a combination of interests of individuals and the State itself. The lack of regulation or inadequate regulation of the resources of the Amazon, and the excesses in their exploitation and indiscriminate use, is a pending problem not only at the national level, but also at the global level.

In the case of Bolivia, legal implementation at the constitutional and legislative levels has been favorable with regard to the protection of natural resources and indigenous peoples. Thus, the aim of the State is “to promote and guarantee the responsible and planned use of natural resources and to promote their industrialization” (Article 9.6). In particular, Title II, entitled “Environment, natural resources, land and territory”, provides for their treatment [70]. In this way, priority is given to the protection of the environment and the multi-ethnic inclusion of those who were not considered for a long time as possessing human dignity.

The new Constitution grants ownership of natural resources to the people of Bolivia and is administered by the State in the collective interest (Article 311.II); the State is responsible for the mineral wealth found in the soil and subsoil (Article 369.I). It also deals with biodiversity, forest resources, hydrocarbons, mining, land, and the Amazon (Articles 390 to 392) [71]. In this context, anyone who violates the constitutional regime of natural resources (Article 124.I) is convicted of treason. According to Juan Antelo, on natural resources (the environment, land, and territory), it is necessary “to formulate, socialize, analyze and constitutionalize the new legal framework in Bolivia, as a prerequisite for regulating the performance of all public and private bodies in this area” [70].

#### 4.3.2. Conflicts Between the Values of Indigenous Peoples and those of Citizens

The internal law of Bolivia (Constitution and Laws) is progressive and supports the rights and recognition of the indigenous worldview. This was reflected in different aspects such as identity, feminism and its struggle for equality and justice among different genders, ethnicities, and social classes, with indigenous communities and their traditions. As a result, the cross-cutting “generic, cultural, ethnic, and generational diversity” and the principles of equity, interculturality and non-discrimination are taken into account throughout the constitutional text [72].

First, indigenous identity is one of the essential elements in order for a human community to be considered a native indigenous rural people and nation (Articles, 30.I). In this context, these peoples enjoy, among other rights to their cultural identity, religious belief, spiritualities, practices and customs, and to their own worldview, so that, if they consider it, the possibility of registering it with Bolivian citizenship and other official identification documents (ethno-citizenship) [73].

Second, is feminism and its struggle for gender equality and justice. The Constitution recognizes the equality of conditions between men and women, as well as a series of guidelines and rights in favor of equality in relation to political participation and internal election of leaders (Articles11.I, 26.I, 147, 210); field of work and wages (Article 48); sexual and reproductive rights (Article 66); vocational and humanistic education (Article 78.IV); and more specific rights directed to women, such as: not to suffer physical, sexual or psychological violence, in the family and in society (Article 15.II); maternity and special care before, during, and after childbirth and in the pre-and post-natal periods (Article 45.V); and in this line, not to be dismissed or discriminated against for any of the above reasons, ensuring their occupational security until the child reaches the age of one (Article 48). Consequently, the State is responsible for promoting policies aimed at eliminating all forms of discrimination against women in access to, tenure in and inheritance of land (Article 402).

With regard to indigenous women, according to PilarUriona, “Living Well” is situated “in the context of the recognition and revaluation of the various existing identities”, with the “challenge to secure a permanently shared political gender agenda so that, in practice, the new legislation reflects the rights and claims that women demand and not the opposite of what is demanded” [74].However, for, Rosario Aquím, the gender proposal, accepted and taken up in its entirety in the current Constitution, “is quite conservative with respect to the expectations that other groups (indigenous, ethnic, homosexual, black, etc.) involved and affected, interested in seeing concrete and profound signs of change.” Consequently, it finds a series of contradictions and advocates “a declaration of principles of its own, on the subject of gender [75].

Third, is sethnic groups and social classes. Discrimination against indigenous people and their populations is a challenge to be overcome throughout the world. In Bolivia, the current Constitution (Article14 and other related articles) eliminates all types of discrimination; especially in 2010, with the entry of the Law against Racism and all forms of discrimination, [64] where Article 15 of the Act prohibits access by indigenous persons to public premises, and instead each public establishment must have a sign stating “All persons are equal before the law.”

#### 4.3.3. Collisions in the Inclusion of Other Minority Groups in Bolivian Law

The ethnic and cultural diversity of the American continent characterizes some ethnic groups whose ancestors lived in Africa. People of African descent remain ethnically and culturally distinct collectives, sharing a common identity, origin, history, and tradition [67]. Afro-descendants living in different parts of the hemisphere, such as Bolivia, call them Afro-Bolivians and Afro-Bolivians. After 183 years of marginalization, these people had their legal recognition in the National Constituent Assembly (2006−2008) [76]. It is a collective that does not identify with indigenous peoples.

The Constitution mentions all the original languages that make up Bolivia, considering the plurinational as part of its commitment to inclusion and social justice, promoting mutual respect and intra-cultural, intercultural and multilingual dialogue (Article 9). The premise is that Bolivia is made up of all Bolivians, the original indigenous peoples, the intercultural, and Afro-Bolivian communities, which together are the people (Article 3).

In order to avoid conflicts, the economic, social, political, and cultural rights of the Afro-Bolivian peoples, as provided for in the Constitution for the native indigenous rural nations and peoples, are recognized in all matters of concern to them. The State also provides them with fiscal land that they do not possess or are insufficient, in accordance with State policy, with a policy of sustainable rural development and women’s ownership of land without discrimination on the basis of their civil status (Article 395).

Finally, with regard to indigenous peoples in danger of extinction, or in voluntary isolation and not contacted [66], their individual and collective lifestyles are protected and respected, and they enjoy the right to continue to maintain their position, the delimitation and legal consolidation of the territory they occupy and inhabit (Article 31).

#### 4.3.4. Social Justice Movements as Agents of Change

Historically, the Bolivian people have stood up in 10 marches against the measures and policies of exclusion implemented by various governments, demanding universal vote, agrarian reform, and the participation of the indigenous majority, women, and farmers in the public and political spheres. Subsequently, other marches have taken place, progressively achieving the inclusion and recognition of the rights of excluded groups, such as the native indigenous peasant populations [21].

The political movement also achieved the legal recognition and inclusion of the Afro-descendant community [76], as well as “the treatment of class, ethnic, and gender exclusion” [74], which were finally incorporated in the Constitution of the State of Bolivia in 2009. According to PilarUriona, the power of social movements “is based on their capacity to handle a discourse of demands of the politics of daily life”, with a form of “mobilization of a horizontal type, that does not respond to an instrumentalization exercised by the dominant groups” [74].

## 5. Conclusions

The vindication of indigenous persons and their peoples in Bolivia has brought important advances in recognition of their rights and dignity, but it has also revived their hope that they will not return to being excluded and oppressed in an unequal society. The question arises as to whether the protection of Bolivian constitutional law and international law in guaranteeing the rights and worldviews of indigenous peoples is sufficient. It is a collective that has been generationally marginalized throughout history, but, through the United Nations Declaration of Indigenous Peoples, significant progress has been made with the recognition of a number of essential rights (collective rights; equality and non-discrimination; self-determination; participation and consultation; free, prior, and informed consent; land and natural resources; development with respect for their culture and identity) [77].

Later, with the American Declaration on the Rights of Indigenous Peoples, it becomes clear that the States themselves are still the natural guarantors of the rights of their citizens, constituting the OAS protection bodies which promote a series of provisions for the promotion and protection of the rights of individuals, who are considered indigenous, and who are identified as such, among their parts. This represents a major step forward in the development of indigenous peoples’ issues and inter-American jurisprudence. In this area, it constitutes an invaluable contribution to the protection of the rights of the indigenous and tribal peoples of the OAS member States. However, although a legal framework, both international and national (constitutional) for the protection of indigenous peoples is fully in place [78], regulatory implementation alone is not enough, but further progress must be made in fulfilling its guarantee in the coming decades, from a multisectoral perspective.

With the adoption of ancestral knowledges (of Living Well “suma qamaña” and Good Life “teko kavi,” among others) State policies have given concrete form to a hopeful process of change toward Living Well. These policies have been incorporated in Bolivian national law, bringing favorable changes and reforms to benefit these populations. Living Well is care for “Mother Earth”, for the person as individual being, social being, collective or group being, and spiritual being, and for the environment. Maintaining the equilibrium of these elements involves harmony within oneself, with one’s environment, and with the cosmos, a plenitude that correlates with health (in a word, well-being). Although approaches to Living Well differ depending on whether one takes an international perspective or an Andean one, there are points of intersection that should be preserved in both dimensions. Specifically, both approaches pursue the protection and guarantee of the rights of indigenous populations, recognizing the unquestionable bond of indigenous populations with their land, their territory, and their natural resources.

As a result, the State must respect the rights of indigenous populations, using the law of prior, free, and informed consultation to guarantee peaceful and harmonious coexistence among indigenous persons, their populations, and the State, as well as equilibrium of the environment and natural resources, such as water. Finally, these rights guarantee quality of life, dignity, health, culture, and preservation of their identity, customs, culture, and oral traditions, among other rights. A long road and many unfinished tasks remain to complete this process, the more so because the process is taking place in a framework conditioned by the individual interests of the States, which sometimes either fail to implement sufficient mechanisms or only do so partially, not taking into account the rights of indigenous populations. But this situation transcends not only our generation and any specific territory, but also the political or economic situation of a single country; it goes beyond even current deforestation of the flora and fauna and the impact on the environment and biodiversity. It involves us all, because if we do not do something to protect these indigenous populations and “Mother Earth” as a whole, there will soon be little we can do. We will have destroyed any glimmer of hope for intergenerational equity.

Finally, changes are not won in a single day. Promoting transformations of such magnitude requires time, courage, monitoring, and rigor to enable mature evaluation and reveal the aspects that need improvement and strengthening. We must also consolidate and continue the work to make the process of change toward Living Well possible, regardless of who comes to power and who dominates the world in any particular circumstances or at any given moment. Therefore, within the framework of the UN Agenda 2030 of SDGs, we must encourage and protect the knowledge of nature and the hereditary knowledge possessed by the rural native indigenous peoples of Bolivia and of other territories. They are an asset of incalculable value for the adoption and success of this crucial global strategy of humanity. This process will be aided by making a breakdown of data and inclusion of an “indigenous identifier” in the UN’s official statistics on countries’ progress toward fulfilment of Agenda 2030.The survival, dignity, wisdom, well-being, and respect of the rights of the indigenous peoples of Bolivia specifically, and of the whole world in general, constitute an essential foundation for successful dialogue between indigenous peoples and States. But above all, they represent hope for our future generations, for respect for our Mother Earth, for sustainable development, and ultimately for our common future on Earth, in the framework of the Andean vision of Living Well.

In the case of the State of Bolivia, its Constitution makes it directly responsible for guaranteeing, protecting, and respecting the rights of indigenous nations and peoples (Article 30.III). It is a good start, that the first guarantor and responsible for a right is the State itself. These policy measures encourage the equal and non-discriminatory treatment of persons, regardless of their original social race, already covered by the Article 2 of the Universal Declaration of Human Rights. However, this does not mean that an ideal situation has been reached in this area. In this country, although the Constitution includes in part different important aspects that have been demanded since the first marches in the last century, there is still much work to be done, not only at the national level, but also at the regional and global levels. The task is not only for the State in question, but for everyone.
